# Clinical and molecular features of resected breast cancer brain metastases: a single center retrospective cohort study

**DOI:** 10.1007/s10549-026-08032-1

**Published:** 2026-07-20

**Authors:** Nira A. Krasnow, Mia Salans, Michelle Jayaraj, Kelsey Kuwahara, Maggie H. Zhou, Lauren Boreta, Steve E. Braunstein, Manish K. Aghi, Jo Chien, Hope Rugo, Michelle E. Melisko, Ramin A. Morshed, Harish N. Vasudevan, Laura A. Huppert

**Affiliations:** 1 Department of Medicine, University of California, San Francisco, CA USA; 2 Department of Radiation Oncology, University of California, San Francisco, CA USA; 3 Division of Hematology/Oncology, Department of Medicine, University of California, San Francisco, CA USA; 4 Department of Neurological Surgery, University of California, San Francisco, CA USA; 5 Division of Breast Medical Oncology, City of Hope Comprehensive Cancer Center, Duarte, CA USA

**Keywords:** Metastatic breast cancer, Brain metastases, Central nervous system metastases

## Abstract

**Purpose:**

To characterize clinical and molecular characteristics and prognosis among patients with metastatic breast cancer (MBC) who undergo brain metastasis (BM) resection.

**Methods:**

We retrospectively identified patients with MBC who underwent BM resection from 2006 to 2024 at a single center. Chart abstraction was utilized to identify key demographic, treatment, and outcome data. Median real-world overall survival (mrwOS) was estimated with the Kaplan-Meier method; the Cox proportional hazards model evaluated factors associated with mrwOS.

**Results:**

107 patients were identified with the following MBC subtypes: hormone receptor-positive (HR+), HER2-negative (HER2-) (*n* = 26, 24.3%), HER2+ (*n* = 45, 42.1%), and triple-negative (TNBC; *n* = 36, 33.6%). At time of initial BM surgical resection, 55 patients (51.4%) had CNS-only disease and 63 patients (58.9%) had a single BM. Most patients received systemic therapy after BM resection (*n* = 83/95 with available systemic therapy records, 87.4%) and postoperative radiation (*n* = 92/103 with available post-operative radiation records, 89.3%). Among patients with available BM receptor status (*n* = 92), 25 (27.2%) had receptor discordance between BM and peripheral tissue testing, mostly loss of BM HR-positivity (*n* = 20/92, 21.7%). Among patients with available BM NGS testing (*n* = 40), the most common pathogenic alterations were *TP53* mutation (*n* = 28, 70.0%), *ERBB2* amplification (*n* = 15, 37.5%), and *PIK3CA* mutation (*n* = 9, 22.5%); 31 patients (77.5%) had therapeutically targetable mutations. mrwOS from BM resection was longer for patients with HR+/HER2- and HER2+ MBC vs. TNBC (40.3 and 37.8 months vs. 12.6 months, *p* = 0.04 and *p* = 0.02 respectively). On multivariate analysis, receipt of systemic therapy (HR 0.09, CI 0.04–0.21, *p* < 0.01) and stereotactic radiosurgery (SRS) (HR 0.41, CI 0.18–0.97, *p* = 0.04) post-operatively were associated with longer mrwOS. Extracranial disease at time of resection (HR 4.60, CI 2.52–8.39, *p* < 0.01) and development of leptomeningeal disease (LMD) (HR 6.08, CI 3.00-12.31, *p* < 0.01) were associated with shorter mrwOS.

**Conclusions:**

Survival after BM resection was ~3 years in patients with HR+/HER2- and HER2+ MBC and ~1 year in patients with TNBC. Extracranial disease at time of BM resection and development of LMD were associated with worse prognosis. Loss of HR-positivity and targetable mutations were present in ~22% and ~75% of resected BMs, respectively, supporting the utility of molecular analysis of resected BMs to guide management.

## Background

Approximately 20–40% of patients with metastatic breast cancer (MBC) develop brain metastases (BM) during the course of their metastatic disease, which is associated with significant morbidity and mortality [1–3]. The incidence of BM in patients with MBC is increasing, in part due to improvements in systemic therapy allowing patients to live longer with metastatic disease as well as advances in BM detection [1, 2]. Surgical resection of breast cancer BMs is often pursued in patients with high performance status, limited intracranial disease, large lesions, and/or symptomatic BMs. However, studies evaluating prognostic factors and survival among this population in a modern cohort are limited.

Prior retrospective studies have described outcomes for patients with MBC and resected BMs. A study of 70 patients with MBC and BMs from 1997 showed a median overall survival of 14 months following neurosurgical resection of breast cancer BM [4]. Another study of ~50 patients from 2013 found a median overall survival of 19 months from surgery, with the presence of multiple systemic metastases and absence of post-operative chemotherapy identified as independent adverse prognostic factors [5]. Surgical and radiation techniques have improved since this time, and both studies predate the expanded use of novel therapeutic agents with intracranial activity, such as tyrosine kinase inhibitors and antibody drug conjugates (ADCs) that are now standard of care [6, 7]. A more recent study examined predictors of survival in 95 patients following breast cancer BM resection between 2008 and 2019, demonstrating a median overall survival of 16 months from BM resection, with ~23% of patients living three or more years [8]. In another study comparing survival between patients with MBC with a single BM versus multiple BMs there was no statistically significant difference identified between the two groups (median survival 17 months and 12.5 months respectively), though they did find adjuvant radiation and trastuzumab treatment were associated with longer post-operative survival in patients with multiple BMs [9]. Additional studies are needed to understand survival and prognostic factors in a modern cohort of patients with MBC who have undergone BM resection.

Though pursued for therapeutic intent, surgical resection of breast cancer BMs also provides a unique opportunity for better understanding the molecular characteristics of central nervous system (CNS) disease. Prior work has demonstrated discordance in receptor subtype status between MBC BM and primary tumors in approximately 40% of patients, which may have important implications for management among patients with intracranial disease [10, 11]. Additionally, widespread availability of next-generation sequencing (NGS) has facilitated the identification of pathogenic alterations in resected BM, with one study finding that over 80% of brain metastases may exhibit unique oncogenic profiles when compared to extracranial disease sites [12]. This genomic heterogeneity offers the potential to identify therapeutic targets in the resected BM specimens, which may offer opportunity to use novel CNS-penetrant targeted agents.

This retrospective cohort study examines treatment characteristics and prognostic factors in patients who underwent resection of breast cancer BM at a single institution. We examined receptor status and pathogenic mutations among resected BMs and compared central versus peripheral receptor and NGS testing as available. We evaluated survival from time of BM resection and identified factors associated with improved prognosis. Ultimately, these results may aid in prognostication and management of patients with resected breast cancer BM and lay the groundwork for future studies incorporating genomic testing results into treatment selection.

## Methods

### Study design and objectives

This is a single-center retrospective cohort study of patients with MBC and BMs who underwent resection of at least one BM at the University of California San Francisco (UCSF) between 2006 and 2024. The primary objective of this study was to define the clinical characteristics and survival outcomes of patients who underwent breast cancer BM resection. Secondary objectives included identification of clinical factors associated with survival and characterization of mutations among resected breast cancer BM.

### Participant identification

Patients were identified for inclusion in this study through searches of the following databases at UCSF: (1) the UCSF Radiation Oncology database for patients with breast cancer undergoing BM radiotherapy, (2) clinical records for patients with resected breast cancer BM, and (3) the UCSF Pathology database for patients with resected breast cancer BM. All patients 18 years of age or older were included. Patients without a pathologically confirmed diagnosis of primary breast cancer or with duplicate entries were excluded on initial screening. Patients with insufficient clinical records (including those with unknown baseline MBC receptor status) or who were lost to follow-up were also excluded.

### Chart abstraction

Patient demographics, treatment information, tumor characteristics, and clinical and survival outcomes were manually extracted from the medical record. These included age at MBC diagnosis, sex, race/ethnicity, presence of active extracranial disease, number of BM at the time of surgery, BM receptor status, NGS results from BM and peripheral tissue, location of the resected index lesion, extent of resection (gross total resection [GTR] versus subtotal resection [STR]), receipt and type of pre- and post-operative systemic therapy, date of surgery, development and dates of local recurrence and/or leptomeningeal disease (LMD), and date of death or last follow-up. Data cutoff for survival analysis was 2/12/2026.

### Statistics

Data were analyzed using Prism Software (GraphPad; San Diego, CA) and Stata v.18 (StataCorp; College Station, Tx). Descriptive statistics were used to summarize patient, tumor, and treatment characteristics as rate of events (%) and median (range) as appropriate. Median real-world overall survival (mrwOS) from the date of BM surgical resection was estimated by the Kaplan-Meier method, and the Cox proportional-hazards model was used to evaluate univariable and multivariable factors associated with mrwOS after brain metastasis resection. Variables of clinical interest and those with a p-value < 0.1 on univariable analysis were subsequently included in the multivariable model. P-values < 0.05 were considered statistically significant. LMD was treated as a time-varying covariate; all other covariates were treated as fixed. Patients with missing data for any of the covariates were excluded from the model.

### Ethical considerations

This retrospective cohort study was approved by the Institutional Review Board at UCSF and was performed in accordance with the Declaration of Helsinki.

## Results

### Patient demographics and BM resection characteristics

107 patients with MBC who underwent BM resection were identified and included in this analysis. Baseline patient characteristics are summarized in Table 1. Most patients were female (*n* = 106, 99.1%), white (*n* = 64, 59.8%), and non-Hispanic (*n* = 89, 83.2%) with a median age at MBC diagnosis of 56 years (range: 26–84 years). Tumors were predominantly of ductal histology (*n* = 86, 80.4%). MBC receptor subtypes at time of MBC diagnosis included: hormone receptor-positive (HR+)/HER2-negative (HER2-) (*n* = 26, 24.3%), HER2+ (*n* = 45, 42.1%), and TNBC (*n* = 36, 33.6%). Nearly 20% of patients (*n* = 21, 19.6%) had *de novo* metastatic disease.

**Table 1:** Patient demographic, breast cancer, and brain metastases characteristics

Characteristic	All patients (*N* = 107)
**Demographics**	
Female sex, no. (%)	106 (99.1)
Age at diagnosis of MBC in years, median (range)	56 (26–84)
Ethnicity, no. (%)	
Hispanic	15 (14.0)
Non-Hispanic	89 (83.2)
Unknown	3 (2.8)
Race, no. (%)	
White	64 (59.8)
Black	8 (7.5)
Asian	16 (15.0)
Native American or Alaska Native	1 (0.9)
Native Hawaiian or Pacific Islander	1 (0.9)
Other	16 (15.0)
Unknown	1 (0.9)
**Breast cancer characteristics**	
Tumor histology, no. (%)	
Ductal	86 (80.4)
Lobular	5 (4.7)
Other/unknown	16 (15.0)
Receptor status of extracranial MBC, no. (%)	
HR+/HER2-	26 (24.3)
HR+/HER2+	20 (18.7)
HR-/HER2+	25 (23.4)
TNBC	36 (33.6)
De novo metastatic disease, no. (%)	21 (19.6)
**Brain metastases characteristics**	
Side of resected BM, no. (%)	
Left	52 (48.6)
Right	55 (51.4)
Location of resected BM, no. (%)	
Frontal	31 (29.0)
Parietal	23 (21.5)
Temporal	9 (8.4)
Occipital	12 (11.2)
Cerebellar	30 (28.0)
Insular	2 (1.9)
Total # BM at time of resection, no. (%)	
1	63 (58.9)
2–4	25 (23.4)
5+	19 (17.8)
Extent of resection, no. (%)	
Gross total resection	83 (77.6)
Sub or near total resection	21 (19.6)
Unknown	3 (2.8)

Abbreviations: Brain metastases (BM); hormone receptor (HR); human epidermal growth factor receptor 2 (HER2); metastatic breast cancer (MBC); number (no.); triple negative breast cancer (TNBC)

Among this cohort of patients who underwent surgical BM resection, most patients had BM present at time of MBC diagnosis (*n* = 65, 60.8%), with a median time from MBC to BM diagnosis of 0 months (range: 0-168.4 months). Most BM resection surgeries occurred within 2 weeks of BM diagnosis (median 0.4 months; range: 0-84.1 months). At the time of BM resection, about half of patients had CNS-only disease without evidence of extracranial disease (*n* = 55, 51.4%) and most (*n* = 63, 58.9%) had a single BM. The most common locations of resected BM included the frontal lobe (*n* = 31, 29.0%), cerebellum (*n* = 30, 28.0%), and parietal lobe (*n* = 23, 21.5%). Most patients (*n* = 83, 77.6%) underwent gross total resection.

### Treatment characteristics

Systemic and radiotherapeutic treatment characteristics are described in Table 2. As stated above, many patients were diagnosed with BM at the time of MBC diagnosis and underwent upfront surgical resection, so only half of patients in this cohort (*n* = 54, 50.5%) received systemic therapy for MBC prior to surgical resection and twenty-six patients (24.3%) received CNS-directed radiation prior to surgery. Among patients with post-operative systemic treatment data available (*n* = 95, 88.8% of cohort), 83 patients received systemic treatment (87.4%). Twelve patients (12.6%) did not receive post-resection systemic therapy for the following reasons: 8 patients enrolled in hospice or died shortly after surgery, 2 patients had stable intracranial and systemic disease so no systemic therapy was recommended, 1 patient had poor functional status so no systemic therapy was recommended, and 1 patient declined systemic therapy. Many patients received post-operative systemic therapies known to have CNS penetration and activity, including HER2-directed tyrosine kinase inhibitors (*n* = 27/95, 28.4%) and trastuzumab deruxtecan (T-DXd) (*n* = 14/95, 14.7%). Among patients with post-operative radiation data available (*n* = 103, 96.3% of cohort), 92 patients (89.3%) underwent postoperative radiation to the resected BM, most often stereotactic radiosurgery (SRS) (*n* = 60, 58.3%) or I-125 brachytherapy (*n* = 14, 13.6%).

**Table 2:** Treatment characteristics

**Systemic and radiation treatments for MBC before BM resection**	***N*** ** = 107**
Receipt of systemic treatment, no. (%)	54 (50.5)
Lines chemotherapy, med (range)	0 (0–6)
Lines ET, med (range)	0 (0–4)
Lines targeted therapy, med (range)	0 (0–2)
Lines total therapy, med (range)	1 (0–8)
Receipt of CNS-active therapies of interest, no. (%)	
HER2-directed TKIs	7 (6.5)
Abemaciclib	1 (0.9)
T-DM1	5 (4.7)
T-DXd	2 (1.9)
Sacituzumab govitecan	1 (0.9)
Immune checkpoint inhibitor	4 (3.7)
Pre-operative radiation to index lesion, no. (%)	26 (24.3)
**Systemic treatments for MBC after BM resection**	**N = 95**
Receipt of systemic treatment, no. (%)	83 (87.4)
Lines chemotherapy, med (range)	1 (0–7)
Lines ET, med (range)	0 (0–3)
Lines targeted therapy, med (range)	0 (0–3)
Lines total therapy, med (range)	1 (0–8)
Receipt of CNS-active therapies of interest, no. (%)	
HER2-directed TKIs	27 (28.4)
Abemaciclib	3 (3.2)
T-DM1	7 (7.4)
T-DXd	14 (14.7)
Sacituzumab govitecan	3 (3.2)
Immune checkpoint inhibitor	8 (8.4)
**Radiation treatment for CNS disease after BM resection**	**N = 103**
Post-operative RT, no. (%)	
Any	92 (89.3)
SRS	60 (58.3)
Partial-brain	8 (7.8)
WBRT	9 (8.4)
I-125	14 (13.6)
Cs-131	1 (1.0)
Post-op RT dose (Gy), med (range)	30.0 (12.0–40.0)
No. post-op RT fractions, med (range)	5 (1–15)
Post-op RT BED (Gy), med (range)	46.9 (26.4–56.0)
Post-op RT PTV (cc) (SRS only), med (range)	8.5 (0.2–31.5)

Abbreviations: Biologically effective dose (BED); brain metastasis (BM); central nervous system (CNS); endocrine therapy (ET); Gray (Gy); human epidermal growth factor receptor 2 (HER2); planning target volume (PTV); radiation therapy (RT), stereotactic radiosurgery (SRS); trastuzumab deruxtecan (T-DXD); trastuzumab emtansine (T-DM1); tyrosine kinase inhibitor (TKI); whole-brain radiation therapy (WBRT)

### Molecular features of resected BM

Molecular characteristics of resected BM, including receptor subtypes and somatic mutation status, are displayed in Table 3. BM receptor subtypes were reported for 92 patients (86.0%); receptor status was not reported on the pathology report for the remaining 15 patients (14.0%). Of the 92 patients with BM receptor status available, 25 patients (27.2%) had a change in receptor status between extracranial MBC tissue and resected BM, including loss of HR (*n* = 20/25 with receptor status change, 80.0%), gain of HR (*n* = 3, 12.0%), gain of HER2 amplification (*n* = 1, 4.0%), or gain of HER2 amplification with loss of HR (*n* = 1, 4.0%). Of the 25 patients with a receptor status change, 6 patients (24.0%) had change in treatment related to the identified change in receptor status, 17 patients (68.0%) did not have a change in treatment related to the subtype change, and 2 patients (8.0%) were unknown due to insufficient post-operative treatment information.

**Table 3:** Molecular characteristics of resected BMs

**Patients with BM receptor status (** *N* ** = 92)**				
	**Overall** **(** *N* **= 92)**	**HR+/HER2- ** **(** *N* ** = 25)**	**HER2+** **(** *N* **= 36)**	**TNBC** **(** *N* **= 31)**
Change in receptor status (extracranial MBC vs. BM), no. (%)				
No change	67 (72.8)	13 (52.0)	23 (63.9)	31 (100.0)
Change	25 (27.2)	12 (48.0)	13 (36.1)	0 (0.0)
Loss HR	20 (80.0)	10 (83.3)	10 (76.9)	0 (0.0)
Loss HR, Gain HER2	1 (4.0)	1 (8.3)	0 (0.0)	0 (0.0)
Gain of HER2	1 (4.0)	1 (8.3)	0 (0.0)	0 (0.0)
Gain of HR	3 (12.0)	0 (0.0)	3 (23.1)	0 (0.0)
**Patients with NGS (** *N* ** = 40)**				
	**Overall** **(** *N* **= 40)**	**HR+/HER2-** **(** *N* **= 11)**	**HER2+** **(** *N* **= 17)**	**TNBC** **(** *N* **= 12)**
Presence of mutation, no. (%)				
ESR1	2 (5.0)	2 (18.2)	0 (0.0)	0 (0.0)
PIK3CA	9 (22.5)	3 (27.3)	6 (35.3)	0 (0.0)
PTEN	5 (12.5)	1 (9.1)	0 (0.0)	4 (33.3)
AKT	2 (5.0)	2 (18.2)	0 (0.0)	0 (0.0)
PALB2	0 (0.0)	0 (0.0)	0 (0.0)	0 (0.0)
BRCA1	6 (15.0)	1 (9.1)	1 (5.9)	4 (33.3)
BRCA2	2 (5.0)	1 (9.1)	0 (0.0)	1 (8.3)
NTRK	0 (0.0)	0 (0.0)	0 (0.0)	0 (0.0)
CDH1	2 (5.0)	1 (9.1)	1 (5.9)	0 (0.0)
TP53	28 (70.0)	5 (45.5)	13 (76.5)	10 (83.3)
ERBB2	15 (37.5)	0 (0.0)	15 (88.2)	0 (0.0)
Presence of targetable mutation^*^	31 (77.5)	8 (72.7)	16 (94.1)	7 (58.3)

^*^ Targetable mutations included ESR1, PIK3CA, PTEN, PALB2, BRCA1, BRCA2, AKT, NTRK, and ERBB2

Abbreviations: Brain metastasis (BM); hormone receptor (HR); human epidermal growth factor receptor 2 (HER2); metastatic breast cancer (MBC); next-generation sequencing (NGS); number (no.); triple negative breast cancer (TNBC)

Next generation sequencing (NGS) for resected BM was available for 40 patients (37.4% of cohort). The most common pathogenic alterations identified on NGS included *TP53* (*n* = 28, 70.0%), *ERBB2* amplification (*n* = 15, 37.5%), *PIK3CA* (*n* = 9, 22.5%), *BRCA1* (*n* = 6, 15.0%), and *PTEN* (*n* = 5, 12.5%). Five patients (4.7%) had both peripheral and BM NGS available, although at different timepoints. Peripheral and BM NGS samples were concordant in two patients (*AKT* mutation present in both BM and extracranial MBC tissue in one patient; *PTEN* and *TP53* present in both BM and extracranial MBC tissue in another patient) and discordant in three patients (one patient with *ESR1* and *TP53* mutations in peripheral MBC tissue but not BM, one patient with *ESR1* mutation in BM but not peripheral MBC tissue, one patient with *ESR1* mutation in peripheral MBC tissue but not BM).

### Local control, CNS progression, leptomeningeal disease (LMD) and overall survival

The median length of CNS imaging follow-up was 17.1 months (range: 0-213.5 months). At time of last follow-up, 22 patients (20.6%) experienced local progression at the site of BM resection, 66 patients (61.7%) experienced any CNS progression, and 22 patients (20.6%) developed LMD. The median times to local progression, any CNS progression, and LMD from BM resection were 7.2 months (range: 0.9–66.7 months), 6.5 months (range: 0-46.6 months) and 7.4 months (range: 0-40.2 months), respectively. Of the 22 patients who developed LMD, 3 patients (13.6%) had HR+/HER2- disease, 11 patients (50.0%) had HER2+ disease, and 8 patients (36.4%) had TNBC.

**Fig. 1:**
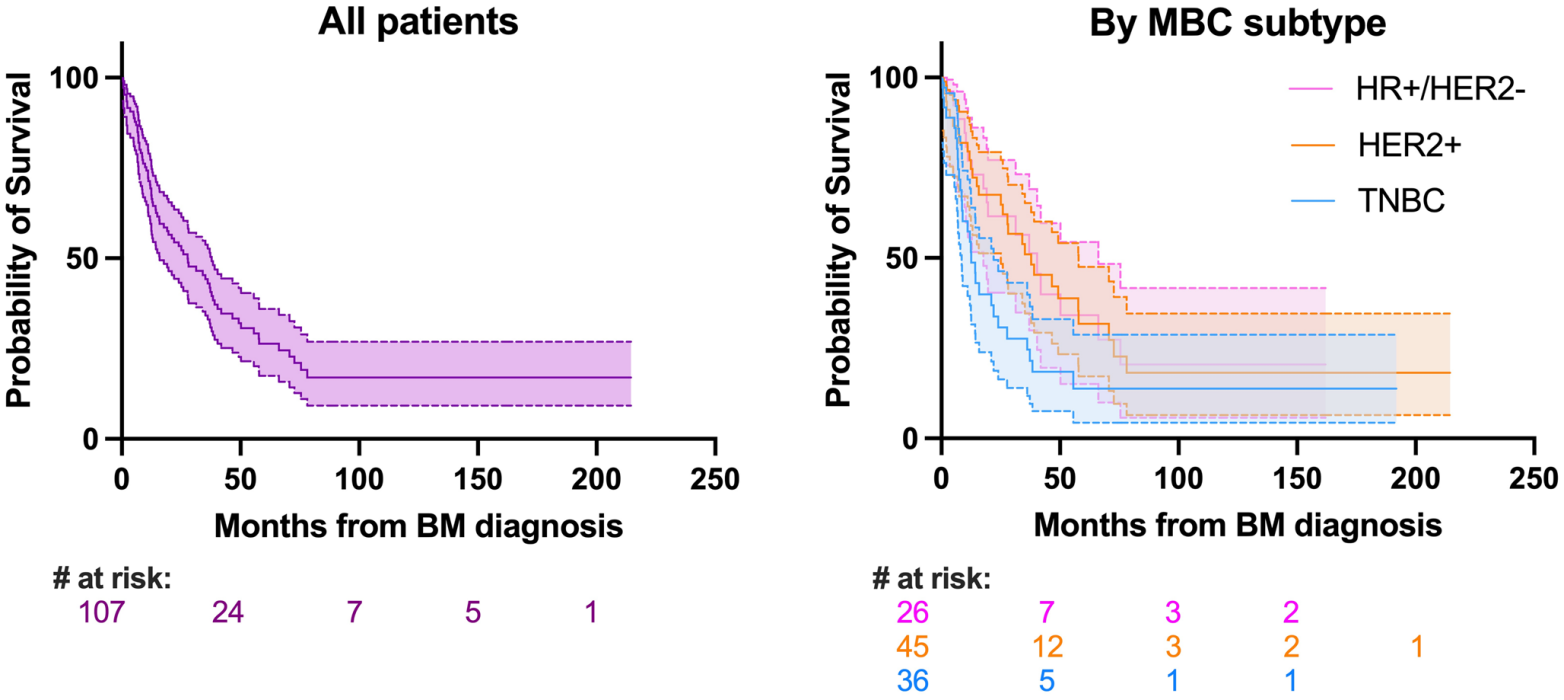
Kaplan-meier survival curves among patients with resected breast cancer BM. Abbreviations: Brain metastasis (BM); hormone receptor (HR); human epidermal growth factor receptor 2 (HER2); metastatic breast cancer (MBC); triple negative breast cancer (TNBC)

mrwOS from BM resection was 27.7 months (interquartile range: 10.4–66.2 months) and differed significantly between patients with HR+/HER2- (40.3 months) and HER2+ (37.8 months) MBC and TNBC (12.6 months) (*p* = 0.039 and *p* = 0.021 respectively) (Fig. 1).

Results of univariable and multivariable analyses of factors associated with mrwOS from time of BM resection are shown in Table 4. For the multivariate analysis, 13 patients had missing data for the included covariates and were excluded; thus 94 patients (87.9% of cohort) were ultimately included. On multivariate analysis, receipt of systemic therapy (HR = 0.09, *p* < 0.001) and stereotactic radiosurgery (SRS) (HR = 0.41, *p* = 0.042) after BM resection were associated with improved mrwOS. The presence of extracranial metastatic disease at time of resection (HR = 4.60, *p* < 0.001) and development of LMD (HR = 6.08, *p* < 0.001) were associated with worse mrwOS.

**Table 4:** Factors associated with overall survival from BM resection to death in univariable and multivariable analysis

Characteristic	Univariable analysis			Multivariable analysis^#^		
	Hazard Ratio	95% CI	*p*-value	Hazard Ratio	95% CI	*p*-value
Age	1.01	1.00-1.03	0.145			
Race White Black Asian Other	Ref 0.51 0.92 0.94	- 0.16–1.63 0.48–1.77 0.51–1.71	- 0.256 0.800 0.828			
Ethnicity Non-Hispanic/Latino Hispanic/Latino	Ref 1.09	- 0.59–2.04	- 0.778			
Metastatic subtype HR+/HER2- HER2+ TNBC	Ref 1.03 1.90	- 0.57–1.88 1.04–3.46	- 0.915 0.037^*^	Ref 0.84 1.33	- 0.42–1.69 0.65–2.75	- 0.633 0.435
Extracranial metastatic disease	2.60	1.62–4.17	< 0.001^*^	4.60	2.52–8.39	< 0.001^*^
Presence of PIK3CA mutation in BM	0.49	0.14–1.67	0.253			
Presence of TP53 mutation in BM	0.58	0.24–1.39	0.221			
Number of BMs 1 2–4 5+	Ref 1.14 1.42	- 0.66–1.97 0.77–2.61	- 0.637 0.257	Ref 1.13 1.03	- 0.58–2.20 0.50–2.16	- 0.719 0.930
Post-operative systemic treatment	0.10	0.05–0.20	< 0.001^*^	0.09	0.04–0.21	< 0.001^*^
Post-operative radiation None WBRT SRS Other	Ref 1.23 0.45 0.69	- 0.46–3.31 0.21–0.96 0.29–1.61	- 0.681 0.040^*^ 0.391	Ref 1.00 0.41 0.81	- 0.31–3.22 0.18–0.97 0.31–2.04	- 0.998 0.042* 0.660
LMD present	4.13	2.44–6.99	< 0.001^*^	6.08	3.00-12.31	< 0.001^*^

^**#**^ All variables of interest and with p-value < 0.1 on univariable analysis were included in multivariable analysis

^*^ Indicates significant at *p* < 0.05

Abbreviations: Brain metastasis (BM); confidence interval (CI); hormone receptor (HR); human epidermal growth factor receptor 2 (HER2); leptomeningeal disease (LMD); stereotactic radiosurgery (SRS); triple negative breast cancer (TNBC); whole brain radiation therapy (WBRT)

## Discussion

Among patients with MBC, BM represent a significant cause of morbidity and mortality. Management of breast cancer BM is complex, typically requiring multimodal therapy, and continues to evolve in the era of novel CNS-penetrant systemic therapies. Patients with BM that are larger in size and/or causing symptoms often require surgical resection, but there remain limited data on outcomes in this population in the modern era. This retrospective cohort study evaluated clinical and molecular characteristics, treatment patterns, and survival among 107 patients with resected breast cancer BM, the largest cohort of patients with MBC and resected BMs to our knowledge.

In our cohort, most patients had HER2+ or TNBC, both of which are subtypes associated with a higher incidence of CNS disease [13]. Approximately 20% had *de novo* MBC and > 60% had BM at time of initial MBC diagnosis, which may be due to delayed diagnosis and larger BM size requiring surgical resection in those with *de novo* or new metastatic diagnoses. At time of BM resection, most patients had disease that was limited to the CNS (*n* = 55, 51.4%) and many had single brain metastases (*n* = 63, 58.9%), which may suggest a preference to pursue surgery in patients with a larger solitary BM, those who have limited systemic disease, and those who have higher performance status. However, 44 patients (41.1%) had more than one BM at the time of resection, including 19 patients (17.8%) with at least five BM. Given that BM resection is typically reserved for patients with limited, symptomatic BM, this may reflect a tendency for more aggressive management of BM at our academic, high-volume institution.

In terms of systemic therapy, many patients received CNS-penetrant therapies before and/or after BM resection. Almost 30% of patients received HER2-directed tyrosine kinase inhibitors such as tucatinib after BM resection, which demonstrated an overall survival benefit when added to capecitabine and trastuzumab among patients with HER2+ MBC with active brain metastases in the HER2CLIMB trial [6]. Only about 15% of patients in this study received post-operative T-DXd, likely due to the fact that many patients were treated prior to the approval of T-DXd for HER2+ and HER2-low/ultralow MBC. T-DXd has demonstrated remarkable efficacy among patients with HER2+ and HER2-ultralow and low MBC, including patients with stable and active BM, so at present most patients with MBC are treated with T-DXd at some point during the course of their metastatic disease, especially in the case of patients with CNS disease [7, 14]. Therefore, it is possible that outcomes would be even better post-BM resection with the use of T-DXd and other brain-penetrant novel targeted therapies and ADCs in the future. Finally, 8% of patients in this cohort received post-operative immune checkpoint inhibitors, which are approved for patients with metastatic TNBC and high PD-L1 expression or patients across subtypes with high tumor mutational burden. Emerging data suggests that checkpoint inhibitors may have some intracranial efficacy as well [15, 16]. 

mrwOS from BM resection in our cohort was 28 months and differed significantly by subtype: patients with HR+/HER2- and HER2+ MBC survived a median of approximately three years post-BM resection, whereas patients with TNBC survived just over one year post-BM resection. These timeframes are longer than those reported in prior studies, which have shown median overall survival of 16–20 months from BM resection [5, 8]. The improvement in survival in this study compared to others is likely in part due to patient selection –– all patients in this cohort underwent surgical resection at a tertiary referral center, thus, the cohort was likely enriched for patients with favorable prognostic characteristics, with access to highly specialized care and clinical trials. Additional factors that may contribute to the survival trends observed include the use of modern surgical and radiation techniques, and/or the increased availability and utilization of CNS-active systemic therapies. On multivariate analysis, extracranial disease at the time of resection and development of LMD were associated with worse prognosis, which is consistent with prior studies [17, 18]. Conversely, treatment with systemic therapy and the receipt of post-operative SRS were associated with improved survival.

In this cohort of patients with resected BMs, we had the unique opportunity to compare breast cancer receptor status and NGS results in the resected BM to systemic tumor pathology results. We observed discordance in receptor status between resected BM and peripheral sites in almost 30% of patients for whom BM receptor subtype data was available (*n* = 92). Loss of HR-positivity was common, occurring in 80% of the 25 patients with receptor discordance. A minority of patients with receptor discordance had gain of HR or HER2 in the resected BM. Less than 25% of patients with receptor discordance had a related change in treatment strategy (*n* = 6/25, 24.0%). Prior studies have shown that change in BM receptor subtype is linked to overall survival, with worse overall survival in patients with loss of ER and a trend towards improved overall survival in patients with HER2 gain, suggesting the importance of BM receptor subtype assessment for both management and prognostication [10]. 

Of the patients in our cohort with NGS of resected BM (*n* = 40), nearly 80% were found to have actionable mutations. Five patients had both peripheral tissue and resected BM tissue available for NGS, three of whom demonstrated discordance between samples. Though limited by small sample size and different timepoints for central and peripheral NGS testing, these results support the utility of pursuing molecular analysis of resected BM as this data may help identify novel therapeutic targets [19, 20]. Further work is needed to better characterize molecular heterogeneity between peripheral and central disease and determine best therapeutic strategies in these cases.

This study has several limitations. First, this was a single-institution study conducted at a high-volume academic center; therefore, the results may not be generalizable to the greater breast cancer population. Second, patients had a variable burden of disease, including number of BM at the time of resection and presence of extracranial disease. As a result, therapeutic goals and management likely varied between patients. Prospective trials that control for these factors are needed to delineate optimal management for patients with breast cancer BM. Third, we did not have a control arm including patients with MBC without BM, limiting our ability to define risk factors for BM development. Nevertheless, we identified characteristics that were enriched in our cohort of patients with resected breast cancer BM unique to this population. This study was also not designed to evaluate whether surgical BM resection is more effective than radiation and/or systemic therapy approaches, so additional studies are needed to address this question, and at present this is often decided on a case-by-case basis depending on pattern of BMs (number, location), patient symptoms, and radiation and/or systemic therapy options for each individual patient. Fourth, not all patients had available BM receptor status (*n* = 92) and BM NGS results (*n* = 40), and only five patients had paired peripheral and BM NGS data. Additional studies that better evaluate concurrent peripheral and central tumor testing may help guide the development of novel treatment paradigms in this patient population. Finally, this study spanned ~20 years to allow for a larger patient cohort, but not all patients received modern surgical, radiation, and systemic therapies; ongoing assessment of survival post-BM resection is needed with further improvements in these therapies in the last few years.

## Conclusions

In summary, this retrospective cohort study defined clinical and treatment characteristics in 107 patients with MBC and BM who underwent surgical BM resection. Median rwOS was more than three years for patients with HR+/HER2- and HER2+ MBC, though worse only one year in patients with TNBC. Receptor subtype discordance between peripheral and BM samples was common, as was the presence of targetable mutations when samples underwent NGS, supporting the utility of molecular characterization of resected BM, though larger, prospective studies are needed to define optimal therapeutic strategies in this patient population.

## Data Availability

The datasets generated during the current study are not publicly available in order to protect patient privacy.

## References

[1] Frisk G, Svensson T, Bäcklund LM, Lidbrink E, Blomqvist P, Smedby KE (2012) Incidence and time trends of brain metastases admissions among breast cancer patients in Sweden. Br J Cancer 106(11):1850–1853. 10.1038/bjc.2012.163 22531629 10.1038/bjc.2012.163 PMC3364124

[2] Darlix A, Louvel G, Fraisse J et al (2019) Impact of breast cancer molecular subtypes on the incidence, kinetics and prognosis of central nervous system metastases in a large multicentre real-life cohort. Br J Cancer 121(12):991–1000. 10.1038/s41416-019-0619-y 31719684 10.1038/s41416-019-0619-y PMC6964671

[3] Lin NU, Claus E, Sohl J, Razzak AR, Arnaout A, Winer EP (2008) Sites of distant recurrence and clinical outcomes in patients with metastatic triple-negative breast cancer: high incidence of central nervous system metastases. Cancer 113(10):2638–2645. 10.1002/cncr.23930 18833576 10.1002/cncr.23930 PMC2835546

[4] Wroński M, Arbit E, McCormick B (1997) Surgical treatment of 70 patients with brain metastases from breast carcinoma. Cancer 80(9):1746–1754. 10.1002/(sici)1097-0142(19971101)80:9&lt;1746::aid-cncr8&gt;3.0.co;2-c 9351543 10.1002/(sici)1097-0142(19971101)80:9<1746::aid-cncr8>3.0.co;2-c

[5] Tabouret E, Metellus P, Tallet-Richard A et al (2013) Surgical resection of brain metastases from breast cancer in the modern era: clinical outcome and prognostic factors. Anticancer Res 33(5):2159–2167 23645770

[6] Murthy RK, Loi S, Okines A et al (2020) Tucatinib, Trastuzumab, and Capecitabine for HER2-Positive Metastatic Breast Cancer. N Engl J Med 382(7):597–609. 10.1056/NEJMoa1914609 31825569 10.1056/NEJMoa1914609

[7] Modi S, Saura C, Yamashita T et al (2020) Trastuzumab Deruxtecan in Previously Treated HER2-Positive Breast Cancer. N Engl J Med 382(7):610–621. 10.1056/NEJMoa1914510 31825192 10.1056/NEJMoa1914510 PMC7458671

[8] Michel A, Darkwah Oppong M, Rauschenbach L et al (2022) Prediction of Short and Long Survival after Surgery for Breast Cancer Brain Metastases. Cancers 14(6):1437. 10.3390/cancers14061437 35326590 10.3390/cancers14061437 PMC8946189

[9] Michel A, Rauschenbach L, Karadachi H et al (2025) Surgical treatment of multiple breast cancer brain metastases: clinical characteristics and factors impacting postoperative survival. J Neurooncol 174(1):157–165. 10.1007/s11060-025-05048-3 40299246 10.1007/s11060-025-05048-3 PMC12198272

[10] Hulsbergen AFC, Claes A, Kavouridis VK et al (2020) Subtype switching in breast cancer brain metastases: a multicenter analysis. Neuro-Oncol 22(8):1173–1181. 10.1093/neuonc/noaa013 31970416 10.1093/neuonc/noaa013 PMC7471502

[11] Kotecha R, Tonse R, Rubens M et al (2021) Systematic review and meta-analysis of breast cancer brain metastasis and primary tumor receptor expression discordance. Neuro-Oncol Adv 3(1):vdab010. 10.1093/noajnl/vdab010 10.1093/noajnl/vdab010 PMC8055057 33898990

[12] Morshed R, Khela H, Nguyen M, PATH-08. MOLECULAR DIVERGENCE RATES DIFFER BETWEEN BRAIN METASTASES AND EXTRACRANIAL DISEASE SITES ACROSS PRIMARY TUMOR HISTOLOGIES et al (2024) Neuro-Oncol 26(Supplement8):viii179–viii179. 10.1093/neuonc/noae165.0707

[13] Kuksis M, Gao Y, Tran W et al (2021) The incidence of brain metastases among patients with metastatic breast cancer: a systematic review and meta-analysis. Neuro-Oncol 23(6):894–904. 10.1093/neuonc/noaa285 33367836 10.1093/neuonc/noaa285 PMC8168821

[14] Harbeck N, Ciruelos E, Jerusalem G et al (2024) Trastuzumab deruxtecan in HER2-positive advanced breast cancer with or without brain metastases: a phase 3b/4 trial. Nat Med 30(12):3717–3727. 10.1038/s41591-024-03261-7 39271844 10.1038/s41591-024-03261-7 PMC11645283

[15] Cortes J, Rugo HS, Cescon DW et al (2022) Pembrolizumab plus Chemotherapy in Advanced Triple-Negative Breast Cancer. N Engl J Med 387(3):217–226. 10.1056/NEJMoa2202809 35857659 10.1056/NEJMoa2202809

[16] Brastianos PK, Kim AE, Giobbie-Hurder A et al (2023) Pembrolizumab in brain metastases of diverse histologies: phase 2 trial results. Nat Med 29(7):1728–1737. 10.1038/s41591-023-02392-7 37268724 10.1038/s41591-023-02392-7 PMC10644912

[17] Zhuang Q, Wong RX, Lian WX, Li YQ, Wong FY (2019) Validation of Modified Breast Graded Prognostic Assessment for breast cancer patients with brain metastases: extra-cranial disease progression is an independent risk factor. Ann Palliat Med 8(4):390–400. 10.21037/apm.2019.02.05 30943738 10.21037/apm.2019.02.05

[18] Znidaric T, Gugic J, Marinko T et al (2019) Breast cancer patients with brain metastases or leptomeningeal disease: 10-year results of a national cohort with validation of prognostic indexes. Breast J 25(6):1117–1125. 10.1111/tbj.13433 31286623 10.1111/tbj.13433

[19] Brastianos PK, Carter SL, Santagata S et al (2015) Genomic Characterization of Brain Metastases Reveals Branched Evolution and Potential Therapeutic Targets. Cancer Discov 5(11):1164–1177. 10.1158/2159-8290.CD-15-0369 26410082 10.1158/2159-8290.CD-15-0369 PMC4916970

[20] Magbanua MJM, Melisko M, Roy R et al (2013) Molecular Profiling of Tumor Cells in Cerebrospinal Fluid and Matched Primary Tumors from Metastatic Breast Cancer Patients with Leptomeningeal Carcinomatosis. Cancer Res 73(23):7134–7143. 10.1158/0008-5472.CAN-13-2051 24142343 10.1158/0008-5472.CAN-13-2051

